# Difference in Subjective Accessibility of On Demand Recall of Visual, Taste, and Olfactory Memories

**DOI:** 10.1155/2018/1630437

**Published:** 2018-01-10

**Authors:** Petr Zach, Petra Zimmelová, Jana Mrzílková, Martina Kutová

**Affiliations:** ^1^Institute of Physiotherapy and Selected Medical Disciplines, Faculty of Health and Social Studies, University of South Bohemia in České Budějovice, České Budějovice, Czech Republic; ^2^Institute of Anatomy, Third Faculty of Medicine, Charles University, Prague, Czech Republic

## Abstract

We present here significant difference in the evocation capability between sensory memories (visual, taste, and olfactory) throughout certain categories of the population. As object for this memory recall we selected French fries that are simple and generally known. From daily life we may intuitively feel that there is much better recall of the visual and auditory memory compared to the taste and olfactory ones. Our results in young (age 12–21 years) mostly females and some males show low capacity for smell and taste memory recall compared to far greater visual memory recall. This situation raises question whether we could train smell and taste memory recall so that it could become similar to visual or auditory ones. In our article we design technique of the volunteers training that could potentially lead to an increase in the capacity of their taste and olfactory memory recollection.

## 1. Introduction

When we ask ourselves or others to close their eyes and imagine how French fries look it is easy task and anyone can do it (at least we presume so). Similarly, to imagine our favorite song or tunes we can do either very well. But what about how French fries smell or how they taste? This is not so easy. Why is there a difference between our four sensory modalities?

Linguistically the term “imagine” may not be appropriate. It is bound to visual mental objects but do we have better ones for acoustic, olfactory, and gustatory memories? We find rarely articles studying the subjective differences between sensory memories availability in the humans. One of these is a study by Schifferstein [1]. In Schifferstein's work participants were asked to “imagine a product or event that elicits a characteristic/conspicuous smell, taste, vision…”. He found that sensory images were more vivid for vision and audition than for smell and taste, but he found no significant difference between vision and audition. Under the term sensory memory availability or recall we understand in our article voluntary act of bringing into the present attention already formed olfactory, gustatory, or visual object from the memory. We presume that anyone can perform such recall without special previous training. But the effect of age, sex, profession, health status, and various other factors may influence it greatly [2, 3]. When working with this memory recall (visual, auditory, gustatory, and olfactory) subject should not be exposed to corresponding sensory organ physical object (French fries) at the time (or at least 2 hours before) of experiment.

*Example of the Visual Pathway*. In order to create visual memory of an object there are at least two cues to happen. The first one depends on the sufficiently lightened external object. Light beams from this object reach the three neuronal layer of retina. Neuronal fibers from there continue to the visual neocortex in the occipital and temporal lobes with the side projection to the lateral geniculate body of the diencephalon [4]. In the case of the evocation of visual memory we presume that temporary reactivation of the previously formed circuits (consisting of not exactly known number of the excitatory and inhibitory synapses) in the neocortex (based on previous formation caused by light beams initiation) leads to the visual imagery of the given object in the mind, the visualization process [5].

*Visual Memory Self-Accessibility*. In order to test the volunteer's ability to bring into his/her attention the visual memory of a previously watched object (memory recall) in the present moment we may ask the volunteer to visualize with the closed eyes for several minutes French fries. Most volunteers would claim they can do this easily. Volunteer can visualize French fries with its color and shape corresponding to regularly looking French fries. This happens more or less without participation of the primary or the secondary visual cortical areas (that are active while seeing an external object with the eyes in the present moment). As a prerequisite for the visualization it is necessary to see French fries many times. In the case of blindness as a birth defect this could not be then tested. Previous formation of French fries visual memory trace requires fully functional optical pathway (the retina, optic nerve and optic tract, radiatio optica, and neocortical visual areas in the brain) [4]. In the case of defect of any part of this circuitry there is no formation of the visual memory imprint for the later visualization on demand. Although visualization of the given object requires a previous visual memory formation via the optical pathway it uses different neuronal circuits whose detailed structure is still a matter of debates.

*Auditory Memory Self-Accessibility*. Similarly to the visual memory recall there is a substantial capability of auditory memory on demand accessibility. Again as in the case of visual memory there is not much difficulty for volunteers to “play” their favorite songs inside their heads that they have listened to many times before. This type of memory recall is exceptionally present in singers, artists, and music composers. Similarly to the visual memory formation, the auditory memory formation happens via auditory pathway terminating in the temporal lobe, gyrus temporalis superior (next to the Wernicke speech center) [6]. In some cases auditory memory on demand recall may yield even better results compared to the visual memory recall (great interindividual differences) [7]. An interesting point is that we do not have a separate word for auditory memory recall similar to visual memory recall (“visualization” but no “auditation”).

*Taste Memory Self-Accessibility*. On the contrary to the visual and auditory memory, the taste memory (although very well developed in the humans, e.g., [8])* is not readily available for on demand recall*, unless there is present taste stimulus. This could be partially explained by one kind of specific poison protective reflex known as conditioned taste aversion reaction [9].

*Olfactory Memory Self-Accessibility*. Similarly to the taste memory recall there are not many options for humans to evoke any smell from the memory on demand unless it happens as a result of the pathological condition (e.g., epileptogenic aura, olfactory hallucinations). The cortical projection of the olfactory path from olfactory bulb, olfactory tract, and striae olfactoriae into the entorhinal, perirhinal cortices and amygdala [10] raises similar question as in the case of the taste memory [11].

## 2. Materials and Methods

We evaluated on demand recovery of the olfactory, taste, and visual memory w/o previous training in three groups of volunteers: group 1 composed of 82 female students volunteers of the South Bohemian University, Czech Republic, age 18–21 years, general nurse/delivery assistant study program, group 2 composed of 38 male/female students (18 males and 20 females) of the Prague Gymnasium, Czech republic, age 12–16 years, and group 3 composed of 31 male/female students (25 females and 6 males) of the South Bohemian University, Czech republic, age 18-19 years, Physiotherapy Study Program. All tasks were carried out either 2 hrs prior to their regular exam so that activation of the sympathetic system was highly probable (group 1) or during lecture (group 2 and 3). All students were given instruction to imagine first smell then taste and lastly image of the French fries for at least 5 minutes each. Then they were asked to write down 0—not possible, 1—minimally, and 2—fully for each of three qualities (smell, taste, and visual). All students were required not to taste or smell relevant objects used in the study at least 2 hrs prior to testing. Data were collected anonymously and analyzed in Excel program. Percentages were graphically presented for each of 3 sensory modalities in all three groups.

### 2.1. Statistical Analysis

In order to find statistically significant difference between sensory modalities and between three groups of subjects we selected analysis of contingency tables by Pearson's chi-squared test. This test was also applied for analysis of gender effect in group 2. Statistically significant results were those with *p* < 0.05. For the statistical analysis was used standard Excel program.

## 3. Results

**Figure 1:**
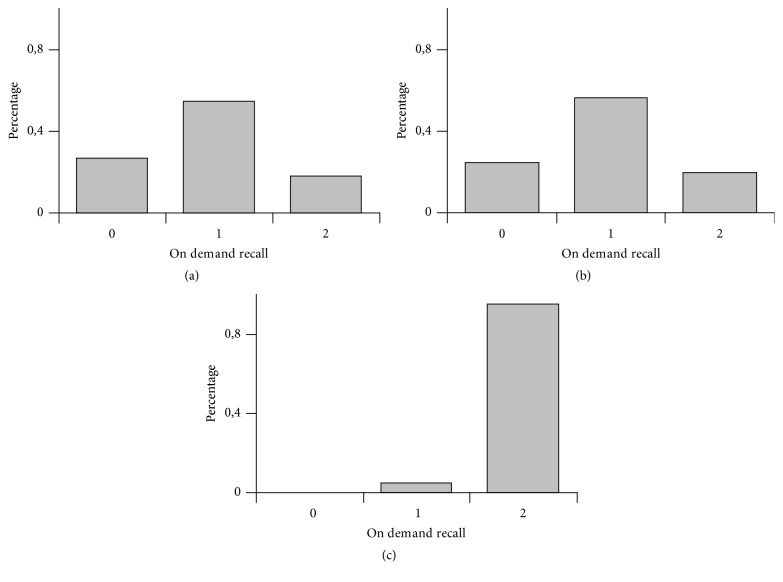
Group 1 (females aged 18–21 years, general nurse/delivery assistants study program). (a) Smell modality, (b) taste modality, and (c) visual modality. On *x*-axis are three categories: 0—not possible, 1—a bit possible, and 2—fully possible. On *y*-axis there are percentages of claims.

**Figure 2:**
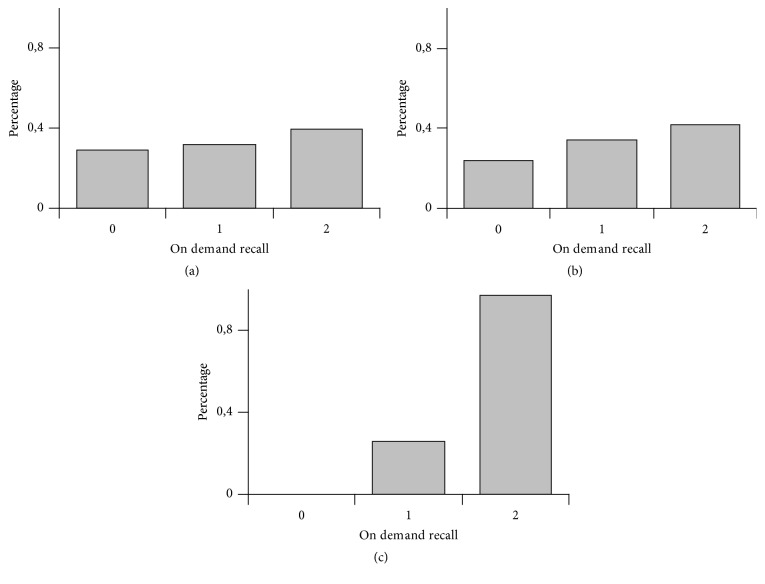
Group 2 (male/female students aged 12–16 years, Gymnasium). (a) Smell modality, (b) taste modality, and (c) visual modality. On *x*-axis there are three categories: 0—not possible, 1—a bit possible, and 2—fully possible. On *y*-axis there are percentages of claims.

**Figure 3:**
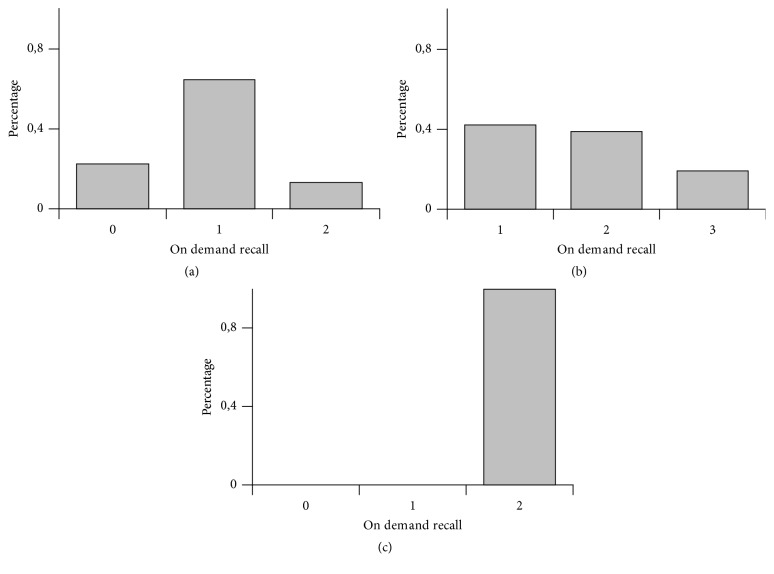
Group 3 (male/female students 18–20 years, Physiotherapy Study Program). (a) Smell modality, (b) taste modality, and (c) visual modality. On *x*-axis there are three categories: 0—not possible, 1—a bit possible, and 2—fully possible. On *y*-axis there are percentages of claims.

For smell memory recovery we observed in group 1 claims 26,8% not possible, 54,9% a bit possible, and 18,3% fully possible (Figure 1(a)), in group 2 claims 28,9% not possible, 31,6% a bit possible, and 39,4% fully possible (Figure 2(a)) and in group 3 claims 22,6% not possible, 64,5% a bit possible, and 12,9% fully possible (Figure 3(a)); for taste memory recovery we observed in group 1 claims 24,4% not possible, 56,1% a bit possible, and 19,5% fully possible (Figure 1(b)), in group 2 claims 23,7% not possible, 34,2% a bit possible, and 42,1% fully possible (Figure 2(b)), and in group 3 claims 41,9% not possible, 38,7% a bit possible, and 19,3% fully possible (Figure 3(b)); for visual memory recovery we observed in group 1 claims 0% not possible, 4,8% a bit possible, and 95,1% fully possible (Figure 1(c)), in group 2 claims 0% not possible, 4,8% a bit possible, and 95,1% fully possible (Figure 2(c)), and in group 3 claims 0% not possible, 0% a bit possible, and 100% fully possible (Figure 3(c)).

### 3.1. Statistics

We tested whether smell, taste, and visual frequency distributions are homogeneous within each group and also whether the three groups gave different answer for each sensory modality. Since groups 1 and 3 consisted mostly of females, we evaluated gender effect of sensory modalities frequency distribution only in group 2. Comparison of smell, taste, and visual modalities showed that visual modality differed significantly from both smell and taste modality in all three groups (*p* < 0.001). On the contrary, smell modality did not differ from the taste modality in all three groups (group 1, *p* = 0.93; group 2, *p* = 0.87; group 3, *p* = 0.12). Effect of gender in group 2 was not significant for any modality (smell, *p* = 0.96; taste, *p* = 0.78; visual, *p* = 0.92).

## 4. Discussion

Taken together we present here theory that humans and maybe also other vertebrates have for not completely clear reasons very good access to visual and auditory memories while very limited access to the taste and olfactory memories. This is also true for the sensitive memory (pain, heat, cold, touch, and vibrations) [12]. It is almost impossible to recall feeling of pain voluntarily (except for pathological states or recent trauma).

The most important aspect of the visualization of the previously seen French fries (probably by activating already formed synapses and dendritic spikes of a particular neuronal circuit) is that* it could be accomplished on demand anytime*. Recently there was an attempt to reconstruct experimentally human visual memory imprints from the brain activity using fMRI [13]. The cinema, for example, combines optical and auditory inputs to the brain that evoke previously formed visual and auditory memory trace. Pathologically, patients may suffer from the visual hallucinations in case of the schizophrenia, physical injury of occipital lobe, and many other diseases.

The most important aspect of the auditory memory recall is that* it could be also accomplished on demand anytime (similarly as in the case of the visual memory recall)*. Pathologically, whistles, noises, sounds, or voices that may be linked to the tumor of the temporal lobe or to the infectious diseases of the brain meningeal coverings or even to the stress effect are more common compared to the visual hallucinations [14].

The reason for unavailability of the taste and olfactory memory recall is not known. Stimulation of the olfactory and taste sensory organs cells usually exerts strong effect on the autonomic system compared to the visual and auditory organs stimulation [15]. This effect on the autonomic system then manifests by increase in the systemic blood pressure or increase or decrease in the plasma hormonal levels. From the social point of view it is greater* faux pas* to lick or smell stranger compared to watch or listen to him/her. The taste and smell sensory organs are also described as the “wet” senses where signal molecule has to chemically interact with the receptor in order to perform its action. On the contrary light beams and acoustic waves have rather an electromagnetic or physical nature. We can easily shield off light signals by closing eyes and sound waves by blocking the ears. But once there is a smell in the air we can not easily avoid it. The hypothalamus is affected more by the taste and olfactory stimuli compared to the optic or acoustic stimuli due to its participation in eating and sexual behavior regulation. Autonomic processes regulation such as blood pressure, temperature, sweating, hunger, satiety, and many others are also more affected by these “wet” sensory organs stimuli compared to visual or auditory ones [16]. Back to the social* faux pas* reaction, the reason behind it is in more profound and inevitable systemic bodily reaction to the “wet” senses stimulation compared to the electromagnetic (visual) or physical (hearing) ones.

If we ask a volunteer (not having the taste stimulus at the present moment) to bring up memory of French fries taste he/she very often fails. In other words: however a volunteer has a very good memory trace of the French fries from the past he/she can not bring it up on demand. This is in contrast with good visual auditory memory recall possibilities. So there is no process like “visualization” in case of on demand taste memory recall although minority of volunteers claim they can do it (Figures 1, 2, and 3). This may have important connection with poisoning by food. The above-mentioned conditioned taste aversion reaction protects from repetitive ingestion of dangerous fruits, but it needs to be started by proper chemical stimulation of the gustatory receptors. If there would be possibility of performing memory recall of such taste event, it could theoretically also trigger binge taste aversion reaction, but at least in humans we do not observe that (at least in literature situations where people would vomit just because of memory recall are not mentioned).

On the other hand, the visual and auditory hallucinations may be linked to the human ability to play with the visual and acoustic memories and to deploy them in the present moment just for fun (in terms of enrichment of the present moment by their memory recall). The taste and olfactory memory could not be similarly integrated in to the present moment. In the case of the olfactory and taste memory accessibility we are almost absolutely dependent on the external stimulation of the sensory organs. This restriction (inability to recall olfactory and taste memories on demand) could be also seen as a way of protection from excessive memory drawback. Spontaneous occurrence of the strong taste memory recall without relevance to present context and without proper stimulus is always considered pathological opposed to the visual or auditory memory recall. Patients undergoing the CT (computerized tomography) scan with intravenously injected contrast matter often experience aberrant taste feelings not attributable to the direct stimulation of the gustatory buds on the mucosa of the oral cavity [17].

Presumption that there is quantitative difference between accessibility of individual modalities of sensory memory gives rise to question: could less accessible memories recall be (gustatory and olfactory ones) trained so that is gets better?

Could it be that hypothetical neocorticalization of an archicortex in the future would grant us the new ability of on demand olfactory memory recall so that there would be “Smell-O-Ramas” similarly to that present day cinemas? Again it would be beneficial to perform the testing and training of the on demand olfactory memory recall in the volunteers. Also people working for many years in the perfume industry could be tested for the olfactory memory recall capability. The possible better memory recall capability in people working in perfume industry could be then differentiated from a better improvement of memory recall capability. Besides the recall of smell, it could be considered to include repeated expositions to the target (French fries). Briefly, experimental subjects would be asked to recall a smell of given kind (e.g., French fries) for 5 minutes twice a day (morning and evening) for the period of at least three months. Then their subjective evaluation of olfactory memory availability should be compared to a control group and tested on fMRI.

We suggest the following protocol for the taste and olfactory memory evocation training experiment: the tested subjects should each day for at least 3 months for 10 minutes in the morning and evening focus mentally on the evocation of some regular food tastes (French fries). After three months of training there should be an evaluation of possible increase in the subjective recall ability compared to the nontrained volunteers.

Subjective testing should be done by the questionnaires to find out subjective levels of the individual memory recall capability in at least 80 age matched subjects in the three age categories: children, mature/productive age, and elderly volunteers. Subjective levels should range from no memory recall (0) to full (2) capability. This testing would be especially yielding in the special groups of sommeliers and olfactory workers, as a third group besides the control one. As a follow up in the cases of possible greater high values in the taste and olfactory memory recall there should be done fMRI scanning focused on the taste and olfactory brain cortices in order to find out hyperactivity and/or activity in the neighboring brain cortical areas. This would be particularly interesting in aforementioned group of sommeliers and olfactory business professionals.

Training of the taste and olfactory memories recall could lead to surprising increase in their capacity and to the enrichment of the personal life. Also it is possible that training of the olfactory memory would decrease the risk of the onset or progression of Alzheimer's disease (the olfactory theory of Alzheimer disease) [18].

## 5. Conclusions

In three studied groups there was better availability of visual memory recall compared to olfactory or taste memory, although there was a bit different percentage on histograms. Further questions arise:

(a) Is the inability to recall taste memory on demand without proper taste stimulus pertinent only to the humans or is it the same in other species?

(b) Did we lose on demand recall of the taste memory during the evolution? Or are we up to it in the future as a result of further brain evolution?

These questions have the same validity in regard to on demand olfactory memory recall. The next question is whether it could be trained or not. This could be tested in sommeliers. If they show a greater capability of on demand taste memory recall it should be then tested on the fMRI to establish which areas of the CNS are differently activated to not trained controls.
